# Distinct roles of early life deprivation and unpredictability in shaping mating-related behaviours and sexual harassment perceptions

**DOI:** 10.3389/fpsyg.2025.1548676

**Published:** 2025-02-25

**Authors:** Chen Xu, Shijin Sun, Xiaoyan Zhang, Zhechen Wang

**Affiliations:** ^1^School of Social Development and Public Policy, Fudan University, Shanghai, China; ^2^School of Humanities, Shanghai Jiao Tong University, Shanghai, China; ^3^School of Psychology, The University of Queensland, Brisbane, QLD, Australia

**Keywords:** early life adversity, reproductive outcome, sociosexual orientation, sexual harassment perception, childhood socioeconomic status, childhood unpredictability, childhood deprivation

## Abstract

**Background:**

Evolutionary-developmental theories propose that early life adversity (ELA) shapes mating patterns. However, evidence is mixed, and the extent to which ELA influences attitudes and perceptions remains underexplored. This research takes a dimensional approach to examine how different forms of ELA relate to mating outcomes and social perceptions in men across two distinct samples.

**Methods:**

Study 1 analysed a U.S. sample (*N* = 1036) using Add Health Wave V data. Study 2 examined a Chinese sample (*N* = 292), collecting data on childhood SES, childhood environmental unpredictability, sociosexual orientation, and perceptions of sexual harassment.

**Results:**

Study 1 found that financial deprivation was associated with a higher number of partner pregnancies and live births but not with the number of sexual partners. Study 2 revealed that childhood unpredictability was positively associated with misperceptions of sexual interest, defined as perceiving mutual romantic interest even when one party experiences unwanted sexual attention. Additionally, childhood SES was positively associated with misperceptions of sexual interest but negatively associated with recognising boundary violations.

**Conclusion:**

These findings suggest that ELA may influence mating-related outcomes through distinct pathways: childhood unpredictability shapes sociosexual orientation, while deprivation-based adversity involves more multifaceted mechanisms, such as cognitive socialisation processes. This research underscores the need for more nuanced predictions within life history theory and highlights the importance of integrating frameworks that account for the cognitive and developmental consequences of deprivation.

## 1 Introduction

Early life adversity (ELA) encompasses a range of adverse experiences, from severe trauma (e.g., abuse) to adverse environmental exposures (e.g., household unpredictability and low socioeconomic status [SES]). Research shows that ELA is associated with health and sexuality-related outcomes. For example, childhood abuse history has been linked to greater sexual risk-taking (Lacelle et al., 2012; Norman et al., 2012; Ramiro et al., 2010), a higher number of sexual partners (Långström and Hanson, 2006; Senn et al., 2006), and sexually coercive behaviours in men (Pedneault et al., 2020).

Previous models of ELA are either specificity models, which focus on individual adversities but overlook their co-occurrence, or cumulative risk models, which count adversities without considering their distinct characteristics (McLaughlin et al., 2021). In contrast, newer dimensional models identified core features shared across adversities. For instance, the Dimensional Model of Adversity and Psychopathology (DMAP) proposes two core dimensions: threat (harm-related adversities, e.g., abuse) and deprivation (lack of resources or stimulation, e.g., material deprivation; McLaughlin et al., 2014). Meanwhile, life history theory, an evolutionary framework that explains how individuals allocate limited bioenergetic resources towards competing biological priorities, identifies ELA dimensions as harshness (threat and deprivation) and unpredictability (unstable and variable environments, e.g., parental transitions and residential moves; Ellis et al., 2009). The life history framework posits that natural selection favours adaptive strategies in which organisms prioritise reproductive development and higher mating effort in response to harsh and unpredictable environments that signal high extrinsic mortality risk (Belsky et al., 1991; Belsky, 2012; Ellis et al., 2009; Figueredo et al., 2007; Stearns, 1992).

An integrated model was recently proposed to incorporate the four facets of adversity (i.e., threat, deprivation, harshness, and unpredictability) to advance the understanding of the distinct effects these core dimensions have on development (Ellis et al., 2022). In the refined framework, three dimensions are emphasised: threat-based harshness, deprivation-based harshness, and environmental unpredictability. Specifically, this model integrated previous dimensional models by dividing harshness into two dimensions: morbidity–mortality caused by harm from others (threat) and morbidity–mortality due to insufficient environmental inputs (deprivation). This distinction is supported by evidence showing that threat and deprivation affect cognitive, emotional, and neural development differently (McLaughlin et al., 2021). For example, a large-scale survey found that threat, but not deprivation, was associated with more risky sexual behaviour, such as having more sexual partners. Indeed, threat-related adversity (e.g., abuse) is consistently linked to earlier reproductive timing and higher offspring numbers (e.g., Yuan et al., 2022).

While threat-related ELA is well-documented in shaping reproductive patterns, the impact of deprivation (e.g., low childhood SES) is less explored and yields mixed findings. Notably, childhood SES is a multifaceted concept encompassing both threat (e.g., direct harm) and deprivation (e.g., limited cognitive stimulation and material insecurity). In industrialised societies, lower SES often reflects relative deprivation, which serves as a proxy for higher mortality risk associated with poverty, rather than absolute deprivation, which constrains the nutritional intake necessary for fertility (Yang et al., 2022). Life history models predict that relative deprivation, but not absolute deprivation, should be associated with a quantity-over-quality reproductive strategy, as absolute deprivation may reduce fertility by limiting the nutritional resources required for reproduction. In this study, we focus specifically on the relative deprivation aspect of childhood SES, emphasising its role as a signal of extrinsic mortality risk. Some studies link low childhood SES to early first births (Xu et al., 2018) and more sexual partners at 16 (McGinnis et al., 2022), while others report no relationship between men's childhood SES and lifetime sexual partners (Xu et al., 2018). Contrarily, some work suggests that higher childhood SES in women predicts more sexual partners, a greater orientation towards short-term mating, fewer long-term partnerships, and fewer offspring (Dinh et al., 2022). This inconsistency highlights the need for a more nuanced exploration of gender-specific effects, as men face lower biological constraints on reproductive effort compared to women.

Unpredictability-based adversity captures environmental instability, such as parental transitions or changes in family structure. Life history theory posits that in unpredictable environments where future mortality is uncertain, prioritising immediate mating opportunities may be an adaptive response (Ellis et al., 2009). This theoretical perspective aligns with findings that family structure changes (e.g., parental absence) predict more sexual partners among males (Xu et al., 2018). In addition, women with fathers who provided low-quality parenting tended to have more sexual partners (Dinh et al., 2022). Higher unpredictability is also linked to more sexual partners at 16 (McGinnis et al., 2022), short-term mating strategies and lower long-term mating orientation (Dinh et al., 2022). While unpredictability is often thought to favour the prioritisation of mating-related effort, it is worth noting that it may also promote bet-hedging strategies, wherein individuals reduce offspring genotypic variance by producing broadly adaptable genotypes or specialised genotypes suited to different environmental conditions (Ellis et al., 2009; Starrfelt and Kokko, 2012; see also Yang et al., 2022). This nuanced perspective underscores the diverse adaptations to unpredictability within life history theory.

In addition to shaping mating behaviours, ELA may influence psychosocial processes, including perceptions of sexual harassment. In the present research, we focus on two key facets of sexual harassment perception: (1) misperceptions of mutual sexual interest, referring to the belief that two individuals share a reciprocal romantic or sexual attraction, even when one party may be uninterested or actively rejecting the other's advances, and (2) perceived harassment, defined as the extent to which individuals identify certain behaviours as violations of social or interpersonal boundaries associated with sexual harassment, including the recognition of actions that overstep norms of appropriate conduct, especially in the workplace or professional settings. ELA may influence these facets by biassing cognitive and perceptual systems to prioritise reproductive opportunities. According to Error Management Theory, men's mating-related perceptions are biassed towards overestimating women's sexual interest to avoid missing potential mating opportunities, which enhances reproductive success (Haselton and Buss, 2000). This bias may manifest as heightened perceptions of sexual intent and reduced recognition of boundary violations in social interactions.

One potential pathway through which ELA shapes these perceptions is via sociosexual orientation, the extent to which individuals pursue unrestricted, short-term mating strategies. The life history framework proposes that harsh and unpredictable childhood may orient individuals towards an unrestricted sociosexual orientation, characterised by a preference for uncommitted sexual relationships (Strouts et al., 2017). This orientation facilitates a quantity-based reproductive strategy, whereby individuals maximise reproductive output through increased mating opportunities. This strategy may be particularly effective for males, who face fewer biological constraints on reproduction than females, whose reproductive output is limited by pregnancy and interbirth intervals (Trivers, 1972). Indeed, evidence suggests that a greater number of sexual partners predicts greater reproductive success in most mammalian males (Janicke et al., 2016), whereas, in females, higher short-term mating orientation is associated with fewer offspring (Dinh et al., 2022). These gender differences support the relevance of focusing on male samples when studying the role of sociosexual orientation, as the positive relationship between sociosexual orientation and reproductive benefits might be more pronounced in men.

Sociosexual orientation may shape perceptions of sexual harassment. While it has been well documented that men are more likely to misperceive sexual interest than women (Fletcher et al., 2014; Perilloux et al., 2012), sex differences become substantially weaker when accounting for sociosexual orientation, suggesting that men's tendency to misperceive sexual interest is partly driven by sex differences in sociosexual orientation (Howell et al., 2012; Lee et al., 2020). In line with Error Management Theory, individuals may interpret sexual interest in ways that correspond to their mating strategies. Supporting this, research shows that sexually unrestricted men perceive greater sexual interest from women (Kohl and Robertson, 2014; Penke and Asendorpf, 2008), and overestimate women's sexual interest (Perilloux et al., 2012). Additionally, because sexually unrestricted individuals perceive less harm in unwanted sexual advances (Klümper and Schwarz, 2020), they may normalise behaviours that others see as boundary violations. Taken together, sociosexual orientation may act as a pathway through which ELA influences men's perceptions of sexual harassment. Specifically, ELA may promote unrestricted sociosexual orientation, which in turn biases perceptions of sexual interest and boundary recognition in social interactions.

The current studies aimed to investigate how two dimensions of ELA—namely, childhood deprivation-based harshness and environmental unpredictability—relate to men's sexual and reproductive outcomes, sociosexual orientation, and sexual harassment perceptions. Given that ELA may impact sexual and reproductive strategies differently for men and women (James et al., 2012; Negriff et al., 2015), this research focused exclusively on men, who face lower reproductive costs and greater variability in mating effort. Both studies are grounded in an evolutionary life history framework, hypothesising that early life harshness and unpredictability are linked to greater mating effort. In particular, Study 1 focuses on behavioural outcomes by investigating whether deprivation-based harshness is associated with increased reproductive effort. Study 2 extends this association by examining perceptual processes, specifically on how deprivation and unpredictability are associated with interpretations of mating-relevant social cues, such as workplace sexual harassment.

## 2 Study 1

The goal of Study 1 was to examine the relationship between retrospective childhood financial deprivation, a proxy for deprivation-based ELA, and mating and reproductive patterns, including the number of partner pregnancies, live births, and lifetime sexual partners, using a nationally representative sample of U.S. men.

### 2.1 Method

#### 2.1.1 Data and participants

National Longitudinal Study of Adolescent to Adult Health (Add Health) is a longitudinal study that followed a nationally representative sample of U.S. adolescents into their adulthood. We used data from the publicly available version of Wave V as the retrospective child financial deprivation question was embedded only in this most recent wave (Harris et al., 2019).

Participants in the original study were between 33 and 43 years old when the fifth wave of data was collected (2016–2018). For the aim of the current study, we limited our analyses to male adults who reported their sexual orientation as 100% heterosexual (*n* = 1,036, *M*_age_ = 38.6, *SD*_age_ = 1.76, Age range = 35–42). In terms of race/ethnicity, the included sample comprised individuals who identified themselves as follows: White (*n* = 778, 75%), African American (*n* = 155, 15%), Hispanic (*n* = 103, 9.9%), Asian (*n* = 37, 3.6%), American Indian (*n* = 32, 3.1%), Pacific Islander (*n* = 7, 0.7%), and other race or origin (*n* = 9, 0.9%). Most participants were married (74%). Their highest education level ranges from high school or lower to a doctoral degree. The R script used to conduct the analyses for Study 1 is accessible at the following link: https://osf.io/rfjt9/.

#### 2.1.2 Measures

##### 2.1.2.1 Sexual and reproductive behaviour

Three variables from the survey were chosen: number of lifetime female sexual partners, times that a partner of yours has been pregnant, and number of live births resulting from these pregnancies. The number of lifetime sexual partners was assessed by asking participants to indicate the number of female sexual partners they have ever had sex with. This also measures the behavioural aspect of sociosexual orientation. Times of partner pregnancy and resulting live births were assessed with with participants reporting how many times a partner of yours has been pregnant, and how many live births resulted from these pregnancies. Descriptive statistics are reported in the results section.

##### 2.1.2.2 Childhood financial deprivation

Childhood financial deprivation was measured by asking participants to indicate whether their family was better off or worse off financially than the average family before the age of 16. They responded on a 5-point scale from 1 (a lot better off) to 5 (a lot worse off).

##### 2.1.2.3 Controlled variables

Age, level of education, current subjective SES, race, and marital status, were included as covariates. We condensed the education attainment variable into 6 categories by combining similar ones from the original 15 categories. Therefore, the level of education was assessed from 1 (some high school or lower) to 6 (doctoral degree). Current subjective SES was measured by asking participants to rate their position on a 10-rung ladder. Marital status was evaluated by querying participants about their current marital status, including options for married, widowed, divorced, separated, or never married. As the majority of the sample were married, to avoid redundancy, we transformed this variable into a binary one, where 0 represented not being married and 1 represented being married. Similarly, race was included as a binary variable, where 1 represented being white and 0 represented all other racial groups.

In addition to including current SES and education as covariates, we explicitly tested their roles as moderators by examining their interactions with childhood financial deprivation in all regression models. All variables included in the interaction analysis were standardised to ensure comparability.

### 2.2 Data analysis

As a preliminary step to the analysis, we tested whether the three count outcome variables were over-dispersed by comparing the variance to the mean for the dependent variables. Overdispersion occurs when the variance of a count variable is substantially greater than its mean (Hilbe, 2011). For the sexual partners variable, the mean was 15.64, and the variance was 415.69, showing substantial overdispersion. For the pregnancy times variable, the mean was 2.65, and the variance was 2.4, indicating under-dispersion. Similarly, for the number of live births variable, the mean was 1.97, and the variance was 1.36, suggesting under-dispersion. Thus, we used Poisson regression to examine the effect of childhood financial deprivation on partner pregnancy counts and resulting live births, and used negative binomial regression for the outcome variable of the number of sexual partners. This is because Poisson regression, which assumes equal mean and variance, becomes inefficient for over-dispersed data where variance exceeds the mean. Negative binomial regression, with an extra parameter to model overdispersion, provides more accurate adjustments and reliable estimates for such data. A zero-inflated Poisson regression model was not estimated since the percentage of zero in three outcome variables was low (0% for lifetime sexual partners, 0% for partner pregnancy times, and 8.7% for resulting live births). Considering the possibility that current education and the present socioeconomic environment in which an individual resides could contrast with their earlier ELA exposures, we sought to investigate whether current education or subjective SES serve as modifying factors. The correlation between education and subjective SES was 0.4, reflecting a moderate relationship that is unlikely to pose multicollinearity concerns. Additionally, we included age, race, and marital status in all regression models to control for their potential effects.

### 2.3 Results

Table 1 summarises the means/proportions and standard deviations among all variables. The number of lifetime sexual partners ranged from 1 to 113, with a mean of 15.6. The number of partner pregnancies ranged from 1 to 10, with 10 indicating 10 or more pregnancies. The cases primarily clustered between 1 and 4 (inclusive), accounting for 89% of the cases. The average number of partner pregnancies is 2.65. Similarly, the number of live births ranged from 0 to 7, with 7 indicating 7 or more childbirths. The cases mostly clustered between 0 and 3 (inclusive), constituting 91% of the total cases, with an average of 1.97.

**Table 1:** Descriptive statistics in Study 1.

**Variables**	**Mean/prop**.	***SD***
**Education**		
Some high school or lower	0.04	
Completed high school or equivalent	0.23	
Community college or equivalent	0.23	
College or equivalent	0.32	
Master's degree	0.14	
Doctoral degree	0.03	
**Marital status**		
Married	0.74	
Not married	0.26	
Age	38.6	1.76
**Race**		
White	0.75	
Other	0.25	
Current subjective SES	5.68	1.93
Childhood financial deprivation	2.96	0.94
Number of lifetime sexual partners	15.6	20.4
Number of partner pregnancies	2.65	1.55
Number of live births	1.97	1.17

Prop., proportion; SD, standard deviation; SES, socioeconomic status. Marital status coded as: 0 = Not Married; 1 = Married.

We then estimated Poisson regression to examine the effect of childhood financial deprivation on the number of partner pregnancies and live births, and estimated negative binominal regression for the outcome variable of lifetime sexual partners. For ease of interpretation, we present results in incidence rate ratios and corresponding confidence intervals in Table 2. When using incidence rate ratios, a value above 1 indicates a positive association and a value below 1 represents a negative association.

**Table 2:** Regression results in Study 1.

	**Number of partner pregnancies**	**Number of live births**	**Number of sexual partners**
	**IRR (CI)**	**IRR (CI)**	**IRR (CI)**
Age	1.01 (0.99–1.03)	1.02 (0.99–1.04)	0.99 (0.96–1.03)
Education	0.98 (0.94–1.02)	0.95 (0.91–1.00)	0.98 (0.91–1.04)
Marital status	1.00 (0.91–1.09)	1.25^***^ (1.12–1.40)	0.50^***^ (0.44–0.58)
Race	0.85^***^ (0.78–0.93)	0.93 (0.84–1.02)	0.81^**^ (0.70–0.92)
Current SES	1.01 (0.97–1.05)	0.99 (0.94–1.04)	1.03 (0.97–1.10)
CFD	1.05^*^ (1.01–1.09)	1.05^*^ (1.01–1.10)	0.99 (0.94–1.06)
CFD^*^Education	1.02 (0.98–1.06)	1.03 (0.98–1.08)	0.96 (0.90–1.02)
CFD^*^Current SES	0.97 (0.93–1.01)	0.98 (0.93–1.02)	0.95 (0.90–1.01)

^*^p < 0.05. ^**^p < 0.01. ^***^p < 0.001. IRR, incidence rate ratio; CI, confidence interval; SES, socioeconomic status; CFD, childhood financial deprivation.

As shown in Table 2, childhood financial deprivation was a significant predictor of the number of partner pregnancies (*p* = 0.012) and the number of live births (*p* = 0.020). Specifically, for every unit transition towards feeling financially deprived in childhood, the incidence rate of partner pregnancies and live births would be expected to increase by 5% and 5.5%, respectively, while holding the other variables in the model constant. However, childhood financial deprivation was not a significant predictor of the number of lifetime sexual partners (*p* = 0.868). Race was negatively associated with partner pregnancy times (*p* < 0.001) and sexual partners (*p* = 0.002) but not the number of live births (*p* = 0.135). These results suggest that being White is associated with decreases in both partner pregnancy times and the number of sexual partners. Marital status was a significant predictor for the number of live births (*p* < 0.001) and sexual partners (*p* < 0.001). Specifically, being in marriage increased the incidence rate of live births by 25%, and decreased the incidence rate of the number of sexual partners by 49.6%. None of the interaction effects were statistically significant, suggesting that the relationship between childhood deprivation and the outcomes does not differ based on education or current SES.

### 2.4 Discussion

In summary, we found some support for the effect of deprivation-related ELA on reproductive outcomes among a group of heterosexual men across a range of ethnicities. This finding partially supports the life history framework, as higher childhood financial deprivation was associated with more partner pregnancies and live births, both of which reflect a quantity-based reproductive strategy. Childhood financial deprivation was not related to the number of lifetime sexual partners, contradicting predictions from life history theory but aligning with findings from a past meta-analysis (Xu et al., 2018). However, financial deprivation was assessed using a single-item measure, which may limit reliability and fail to capture the full scope of early life deprivation. This is an inherent limitation of using existing datasets. Notably, the meta-analysis found that parental absence in childhood, rather than family SES, was robustly associated with the number of sexual partners in men. The present findings underscore the importance of disentangling the nuanced patterns by which different dimensions of ELA are linked to reproductive and sexual behaviours.

## 3 Study 2

Study 2 aimed to investigate how two dimensions of ELA, childhood SES (as a proxy for deprivation-based adversity) and environmental unpredictability, affect men's sociosexual orientation and their perceptions of sexual harassment. To account for the distinct effects of these dimensions, we examined their independent contributions to mating-related outcomes. Drawing on the life history framework, which predicts that higher ELA leads to increased reproductive and mating efforts, we hypothesised that men with greater ELA would exhibit a more unrestricted sociosexual orientation. Additionally, based on Error Management Theory (Haselton and Buss, 2000), which posits that men's perceptions of sexual interest are biassed to maximise reproductive opportunities, we further hypothesised that experiencing ELA would be more likely to perceive higher mutual interest and less likely to recognise behaviours constituting sexual harassment. Finally, we examined whether sociosexual orientation mediates the relationship between ELA and perceptions of sexual harassment.

### 3.1 Method

#### 3.1.1 Research ethics and open practices

The present study was approved by the Institutional Review Board, School of Social Development and Public Policy, Fudan University (approval number: FDU-SSDPP-IRB-2023-1-049). All participants provided informed consent by checking an onscreen box to confirm that they had fully understood the implications of participation and their right to withdraw at any point.

All data and R script are available in OSF: https://osf.io/rfjt9/.

#### 3.1.2 Participants

We used G^*^Power to estimate the sample size (Faul et al., 2007). The sample size was 244 for a small-to-medium effect of Cohen's *f*^2^ = 0.05 which could be detected with alpha = 0.05 and power = 0.80. We recruited an initial sample of 305 participants from the online crowd-sourcing platform Credamo (http://www.credamo.com). Participants met the screening criteria including being over 18 years old, being a heterosexual man, and having a prior Credamo task approval rating of no less than < 80%. At the beginning of the survey, participants read a brief introduction about survey procedures and provided informed consent. An attention check question (e.g., please choose the answer “not at all”) was embedded in the questionnaire to ensure that participants answered carefully. Data of participants who failed the attention check were not recorded. In addition, we manually removed data of 13 participants who identified themselves as a woman (*n* = 2), bisexual (*n* = 5), and homosexual (*n* = 6) because they failed to meet the eligibility requirements. The final sample consisted of 292 heterosexual male participants (*M*_age_ = 31.66, *SD*_age_ = 8.12, Age range = 18–62). Regarding the educational level (1 = primary school or under, 2 = junior high school, 3 = high school, 4 = bachelor's degree or equivalent, and 5 = master's degree or above), 0.3% (*n* = 1) had no more than primary school education, 1.0% (*n* = 3) had junior high school education, 4.1% (*n* = 12) had high school education, 82.5% (*n* = 241) had a bachelor's degree or equivalent, and 12.0% (*n* = 35) had a master's degree or higher. As for their relationship status, 16.1% (*n* = 47) were single, 4.8% (*n* = 14) were in a short-term relationship for < 6 months, 12.7% (*n* = 37) were in a long-term relationship for more than 6 months, and 66.4% (*n* = 194) were married.

#### 3.1.3 Procedure

Participants first provided demographic information. Next, they completed a series of self-report measures. They began by reading a hypothetical workplace sexual harassment scenario and rated the extent to which they perceived sexual harassment within it. Subsequently, participants reported their sociosexual orientation, childhood environmental unpredictability, and childhood economic status.

Although we also collected data on life history strategy and sexual harassment proclivity using established scales, we chose not to include these variables in the formal analyses due to potential social desirability bias and recent critiques of the validity of these measures (Gruijters and Fleuren, 2018; Mededović, 2020).^1^

#### 3.1.4 Materials and measures

##### 3.1.4.1 Sexual harassment perception

We assessed sexual harassment perception using an adapted measurement from Shi and Zheng (2021). Participants read a hypothetical scenario involving a male coworker, Xiaotao, who directed persistent, unwanted romantic attention towards a female colleague, Xiaoyun. The scenario read:

“Xiaoyun is a female employee of a company, sensitive to the needs of others, shy, yielding, and refraining from harsh language. Xiaotao is a male coworker of Xiaoyun. They sometimes discuss issues related to work, performance, and the future of the industry, but have no interactions outside work and are not friends. Recently, Xiaotao began to repeatedly pursue Xiaoyun. Although she had stated several times that she did not want to develop a romantic relationship with him, Xiaotao did not give up, insisting on showing his love and finding opportunities to be alone with her.”

After reading the scenario, participants responded to five items assessing their perception of whether sexual harassment occurred in this situation: (a) “Xiaoyun wanted Xiaotao's attention,” (b) “Xiaoyun would like to receive such attention from Xiaotao,” (c) “Xiaoyun enjoyed Xiaotao's words and deeds,” (d) “Xiaotao violated Xiaoyun's rights,” and (e) “Xiaoyun was sexually harassed by Xiaotao.” Participants responded to these items on a 7-point Likert-type scale from 1 (*strongly disagree*) to 7 (*strongly agree*). A principal axis factor analysis identified two factors: *Misperceptions of Sexual Interest* (first three items; Cronbach's α = 0.87) and *Perceived Harassment* (last two items; Cronbach's α = 0.81). To calculate the overall sexual harassment perception score, the *Misperceptions of Sexual Interest* items were reverse-coded, and all five items were averaged (Cronbach's α = 0.79), with higher scores indicating a stronger perception of unwanted sexual attention.

##### 3.1.4.2 Sociosexual orientation

We used the Sociosexual Orientation Inventory-Revised (SOI-R, Penke and Asendorpf, 2008) to assess one's attitudes, desires and behaviours towards having uncommitted sex, namely, one's sociosexual orientation. This SOI-R was a revised version of the classical measure of the Sociosexual Orientation Inventory (SOI), and the Chinese version of SOI-R was used in our study (which can be downloaded at http://www.larspenke.eu/en/research/soi-r.html). The 9-item SOI-R consists of three subscales: (1) SOI-R behaviour (3 items, Cronbach's α = 0.83), e.g., “With how many different partners have you had sex within the past 12 months?” (from 1= 0 to 9 = 20 or more); (2) SOI-R attitudes (3 items, Cronbach's α = 0.82), e.g., “Sex without love is OK.” (from 1 = *strongly disagree* to 9 = *strongly agree*); (3) SOI-R desire (3 items, Cronbach's α = 0.92), e.g., “How often do you have fantasies about having sex with someone you are NOT in a committed romantic relationship with?” (from 1 = *never* to 9 = *at least once a day*). The full scale also showed good internal consistency (Cronbach's α = 0.90). Items for each subscale were averaged to represent the behaviour, attitudes, and desire aspects of sociosexual orientation. In addition, all nine items were averaged to generate an overall score for sociosexual orientation, with a higher score indicating a more unrestricted sociosexual orientation.

##### 3.1.4.3 Childhood environmental unpredictability

We used an 8-item scale adapted from Young et al. (2018) to assess participants' subjective childhood environmental unpredictability. The first three items were developed by Mittal et al. (2015; Cronbach's α = 0.62 in the original study), and Young et al. (2018) added five items to the original version to increase the measure's reliability. The 8-item scale demonstrated excellent internal consistency (Cronbach's α = 0.92 in the original study), and principal axis factor analysis showed that the items were loaded on a single factor (Young et al., 2018). In the present study, we translated eight items into simplified Chinese and had another researcher translate them back to compare the two versions. Sample items were “My family life was generally inconsistent and unpredictable from day to day” and “My parent(s) frequently had arguments or fights with each other or other people in my childhood.” Participants were instructed to “think back to your life when you were younger than 14” and then responded to items on a 7-point Likert-typed scale from 1 (*not at all*) to 7 (*extremely*). These items were then averaged (Cronbach's α = 0.91), with a higher score indicating more exposure to environmental unpredictability during childhood.

##### 3.1.4.4 Childhood SES

We used a 4-item scale adapted from Griskevicius et al. (2011) to assess participants' retrospective childhood SES. The Chinese version of this measure was frequently used with Chinese samples and showed acceptable internal consistency, such as Wang and Chen (2016) study (Cronbach's α = 0.77 in the original study). Sample items were “My family usually had enough money for things when I was growing up,” and “I grew up in a relatively wealthy neighbourhood.” Participants in the current study responded to items on a 7-point Likert-typed scale from 1 (*strongly disagree*) to 7 (*strongly agree*). SES was indicated by an average score of the four items (Cronbach's α = 0.94), with a higher score showing a more affluent childhood economic status.

### 3.2 Results

#### 3.2.1 Preliminary analyses and descriptive statistics

Table 3 presents means, standard deviations, and correlations for all variables.

**Table 3:** Descriptive statistics and correlations among variables in Study 2.

**Variables**	***M (SD)***	**1**	**2**	**3**	**4**	**5**	**6**	**7**	**8**	**9**	**10**
1. Childhood unpredictability	2.14 (1.08)	–									
2. Childhood SES	4.08 (1.62)	−0.35^***^	–								
3. SOI-R attitude	3.22 (1.91)	0.38^***^	−0.18^**^	–							
4. SOI-R desire	2.32 (1.40)	0.32^***^	−0.11	0.64^***^	–						
5. SOI-R behaviour	1.91 (0.96)	0.26^***^	−0.06	0.65^***^	0.61^***^	–					
6. SOI-R	2.48 (1.25)	0.38^***^	−0.15^*^	0.92^***^	0.86^***^	0.82^***^	–				
7. Perceptions of sexual interest	2.02 (1.1)	0.14^*^	0.06	0.16^**^	0.18^**^	0.15^**^	0.19^**^	–			
8. Perceived harassment	4.49 (1.59)	−0.02	0.11	−0.04	−0.07	−0.06	−0.06	−0.37^***^	–		
9. Sexual harassment perception	5.38 (1.08)	−0.10	0.03	−0.12^*^	−0.15^*^	−0.13^*^	−0.15^**^	−0.84^***^	0.82^***^	–	
10. Age	31.66 (8.12)	−0.11	0.05	−0.1	−0.12^*^	0.04	−0.08	−0.01	−0.06	−0.03	–
11. Education	4.05 (0.48)	−0.06	0.07	0.01	0.01	−0.04	0.00	−0.03	−0.05	−0.01	−0.19^***^

^*^p < 0.05. ^**^p < 0.01. ^***^p < 0.001. SOI-R, sociosexual orientation; SES, socioeconomic status.

First, we examined whether sexual harassment perception varied by age, education, or relationship status. Sexual harassment perception measures were not correlated with age or education. In terms of relationship status, a one-way ANOVA revealed significant differences based on relationship status. Given that the assumption of equal variances was violated (*p* = 0.012), we used *Welch's F* test and the Games-Howell for *post hoc* analysis. Results showed that participants' perception of sexual harassment differed by relationship status, *F*_(3, 46.70)_ = 4.53, *p* = 0.007, ω^2^ = 0.04. Specifically, participants who were single or in long-term relationships had higher perceptions of sexual harassment than those who were married.

Next, we conducted a principal axis factor analysis on the 5 items measuring sexual harassment perception, using oblique rotation (direct oblimin). The sampling adequacy was supported (KMO = 0.73), and Bartlett's Test of Sphericity was significant (*p* < 0.001), supporting the suitability of factor analysis. This analysis yielded a two-factor solution based on eigenvalues > 1, explaining 81.3% of the variance. The first factor, “misperceptions of sexual interest” (items 1–3; e.g., “Xiaoyun wanted Xiaotao's attention”), had an internal consistency reliability of 0.87, with higher scores indicating higher perceived sexual interest. The second factor, “perceived harassment” (items 4–5; e.g., “Xiaotao violated Xiaoyun's rights”), had a reliability of 0.81, with higher scores indicating higher perceived harassment.

#### 3.2.2 Associations between ELA and sexual harassment perception

As shown in Table 3, childhood unpredictability was positively correlated with unrestricted sociosexual orientation and misperceptions of sexual interest, but not with perceived harassment. Childhood SES was negatively correlated with an unrestricted sociosexual orientation but was not correlated with any of the sexual harassment perception variables. Additionally, childhood SES did not correlate with the behavioural subscale of sociosexual orientation (e.g., number of sexual partners), consistent with findings from Study 1.

We then conducted hierarchical multiple regressions to examine how childhood environmental predictability, childhood SES, and sociosexual orientation are associated with perceptions of sexual harassment (i.e., misperceptions of sexual interest, perceived harassment, and overall sexual harassment perception). In Step 1, demographic covariates (age, education, and relationship status) were entered to control for potential confounds. Relationship status was dummy-coded, with “married” being the reference level. In Step 2, childhood unpredictability and SES were included to examine their unique associations with perceptions of sexual harassment. Step 3 introduced sociosexual orientation, given its established links to cognitive biases related to mating and sexual intent (Perilloux et al., 2012; Kohl and Robertson, 2014; Klümper and Schwarz, 2020). The analysis aimed to address two key objectives: (1) to determine if the effects of unpredictability and SES on sexual harassment perceptions are robust to the inclusion of sociosexual orientation, and (2) to assess the unique contribution of sociosexual orientation as a predictor of sexual harassment perceptions. This approach allows for a more nuanced understanding of whether the effects of unpredictability and SES are direct or potentially mediated through sociosexual orientation. The results of the hierarchical regression analyses are summarised in Table 4.

**Table 4:** Hierarchical regression results in Study 2.

	**Model 1**					**Model 2**					**Model 3**				
**Predictors**	***b***	β	***t*** **(*****p*****)**	*R* ^2^	***F*** **(*****p*****)**	***b***	β	***t*** **(*****p*****)**	Δ*R*^2^	Δ***F*** **(*****p*****)**	***b***	β	***t*** **(*****p*****)**	Δ*R*^2^	Δ***F*** **(*****p*****)**
**(A) Perceptions of sexual interest (mean of items 1–3)**															
Age	−0.01	−0.07	−0.92 (0.359)	0.04	2.10 (0.065)	−0.01	−0.06	−0.78(0.437)	0.03	5.27 (0.006)	−0.01	−0.06	−0.80 (0.422)	0.02	4.8 (0.029)
Education	−0.05	−0.02	−0.39 (0.701)			−0.04	−0.02	−0.28 (0.779)			−0.05	−0.02	−0.35 (0.730)		
Single	−0.34	−0.11	−1.63 (0.103)			−0.37	−0.12	−1.81 (0.071)			−0.39	−0.13	−1.89 (0.060)		
Short-term	0.57	0.11	1.80 (0.074)			0.57	0.11	1.78 (0.076)			0.44	0.09	1.37 (0.171)		
Long-term	−0.36	−0.11	−1.67 (0.096)			−0.37	−0.11	−1.76 (0.080)			−0.38	−0.11	−1.77 (0.078)		
CEU						0.20	0.19	3.10 (0.002)			0.14	0.14	2.16 (0.032)		
CSES						0.08	0.12	1.91 (0.058)			0.08	0.12	1.94 (0.054)		
SOI-R											0.12	0.14	2.19 (0.029)		
**(B) Perceived harassment (mean of items 4–5)**															
Age	0.01	0.05	0.76 (0.449)	0.05	2.72 (0.020)	0.01	0.06	0.87 (0.388)	0.02	3.20 (0.042)	0.01	0.06	0.88 (0.383)	0.00	1.16 (0.283)
Education	−0.20	−0.06	−1.05 (0.297)			−0.24	−0.07	−1.25 (0.211)			−0.24	−0.07	−1.22 (0.222)		
Single	0.82	0.19	2.77 (0.006)			0.92	0.21	3.08 (0.002)			0.92	0.21	3.11 (0.002)		
Short-term	0.18	0.02	0.40 (0.692)			0.32	0.04	0.71 (0.481)			0.41	0.06	0.87 (0.376)		
Long-term	0.82	0.17	2.68 (0.008)			0.92	0.19	3.00 (0.003)			0.92	0.19	3.01 (0.003)		
CEU						−0.02	−0.01	−0.22 (0.826)			0.02	0.01	0.17 (0.866)		
CSES						0.14	0.14	2.31 (0.021)			0.14	0.14	2.31 (0.022)		
SOI-R											−0.09	−0.07	−1.08 (0.283)		
**(C) Sexual harassment perception (mean of items 1–5, with items 1–3 reverse-coded)**															
Age	0.01	0.07	1.02 (0.309)	0.05	2.85 (0.016)	0.01	0.07	0.99 (0.324)	0.02	2.47 (0.086)	0.01	0.07	1.01 (0.312)	0.01	3.93 (0.048)
Education	−0.05	−0.02	−0.38 (0.705)			−0.07	−0.03	−0.57 (0.571)			−0.07	−0.03	−0.51 (0.608)		
Single	0.53	0.18	2.65 (0.008)			0.59	0.20	2.93 (0.004)			0.60	0.21	3.00 (0.003)		
Short-term	−0.27	−0.05	−0.88 (0.380)			−0.21	−0.04	−0.68 (0.498)			−0.10	−0.02	−0.32 (0.751)		
Long-term	0.54	0.17	2.62 (0.009)			0.59	0.18	2.85 (0.005)			0.59	0.18	2.87 (0.005)		
CEU						−0.13	−0.13	−2.04 (0.043)			−0.08	−0.08	−1.23 (0.222)		
CSES						0.01	0.01	0.19 (0.846)			0.01	0.01	−0.18 (0.857)		
SOI-R											−0.11	−0.12	−1.98 (0.048)		

SOI-R, sociosexual orientation; CEU, childhood environmental unpredictability; CSES, childhood socioeconomic status.

For misperceptions of sexual interest (Table 4A), the model including only demographic variables was non-significant (*p* = 0.065). In Step 2, adding unpredictability and SES explained an additional 3% of the variance (*p* = 0.006). Childhood unpredictability was positively associated with misperceptions of sexual interest (β = 0.19, *p* = 0.002), while SES was a marginally significant predictor (β = 0.12, *p* = 0.058). In Step 3, the inclusion of sociosexual orientation (β = 0.14, *p* = 0.029) explained a further 2% of the variance (*p* = 0.029). The coefficient for SES remained marginal (β = 0.12, *p* = 0.054), while the effect of unpredictability was reduced (β = 0.14, *p* = 0.032), suggesting that part of the influence of unpredictability on misperceptions of sexual interest may operate through sociosexual orientation. These results indicate that individuals from unpredictable childhoods may be more likely to interpret unwanted sexual attention as involving mutual romantic interest, possibly through the development of a more unrestricted sociosexual orientation.

For perceived harassment (Table 4B), the initial model (Step 1) explained 5% of the variance in perceived harassment (*p* = 0.020), with being single (β = 0.19, *p* = 0.006) and being in a long-term relationship (β = 0.17, *p* = 0.008) emerging as significant predictors. In Step 2, SES was positively associated with perceived harassment (β = 0.14, *p* = 0.021), accounting for an additional 2% of the variance (*p* = 0.042), while unpredictability was not a significant predictor (β = −0.01, *p* = 0.826). In Step 3, the inclusion of sociosexual orientation did not explain additional variance (*p* = 0.283), and its coefficient was non-significant (β = −0.07, *p* = 0.283). SES remained a significant predictor (β = 0.14, *p* = 0.022) after controlling for sociosexual orientation. This suggests that higher childhood SES is linked to greater sensitivity in identifying boundary violations, independent of mating-related sociosexual strategies.

The overall sexual harassment perception score (calculated as the mean of misperceptions of sexual interest and perceived harassment, with misperceptions of sexual interest reverse-coded) was regressed using the same three-step approach (Table 4C). The model with demographic variables was significant (*p* = 0.016), and the inclusion of the second block did not account for additional variance in the outcome variable (*p* = 0.086). In Step 2, childhood unpredictability, but not SES, was a significant predictor (β = −0.13, *p* = 0.043). Adding sociosexual orientation in Step 3 contributed an additional 1% of the variance (*p* = 0.048), and the coefficient of unpredictability reduced (β = −0.08, *p* = 0.222) and was non-significant. This suggests that the influence of childhood unpredictability on the overall measure of sexual harassment perception may operate, in part, through sociosexual orientation.

The regression analyses suggested that the association between childhood unpredictability and sexual harassment perception, as well as the potential mediating role of sociosexual orientation, was primarily driven by misperceptions of sexual interest, as unpredictability did not significantly predict boundary violations. Accordingly, the following path analysis focused on misperceptions of sexual interest. Although sociosexual orientation did not appear to mediate the relationship between SES and perception measures (as the coefficients for SES remained consistent after controlling for sociosexual orientation), SES was included in the path model to account for its shared variance with unpredictability.

#### 3.2.3 Path analysis

**Figure 1:**
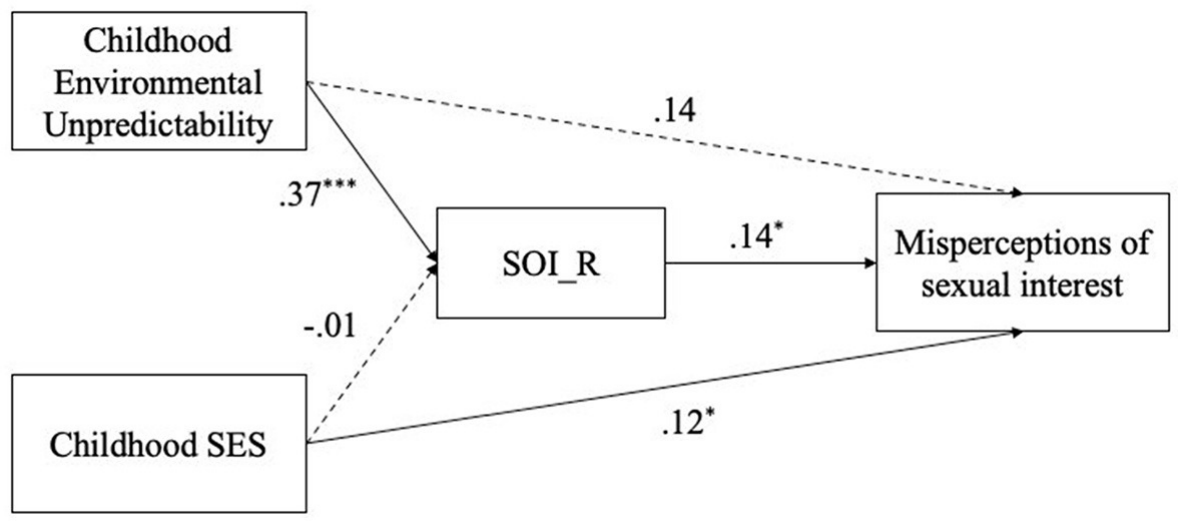
Path model of variables of interest linking to misperceptions of sexual interest. Solid paths indicate significant effects and dashed paths denote non-significant effects. Values reflect standardized regression coefficients. *p < 0.05. ***p < 0.001. SOI_R, sociosexual orientation; SES, socioeconomic status.

To investigate the potential pathways linking early life unpredictability and childhood SES to sexual harassment perception via sociosexual orientation, we conducted a path analysis using the “lavaan” package in R and used 5,000 bootstrap resamples to generate 95% confidence intervals (CIs). Age, education, and relationship status were controlled in the model. Results showed that sociosexual orientation mediated the relationship between childhood unpredictability and misperceptions of sexual interest. Figure 1 presents all standardised direct effects within the path model with misperceptions of sexual interest as the outcome variable.

For the pathway from childhood unpredictability to misperceptions of sexual interest, mediated by sociosexual orientation, childhood unpredictability was positively associated with unrestricted sociosexual orientation (*p* < 0.001), *b* = 0.422, SE = 0.079, 95% CI [0.269, 0.578], and unrestricted sociosexual orientation, in turn, predicted higher misperceptions of sexual interest (*p* = 0.043), *b* = 0.122, SE = 0.060, 95% CI [0.006, 0.242]. The direct effect of childhood unpredictability on misperceptions of sexual interest was significant (*p* = 0.052), *b* = 0.145, SE = 0.075, 95% CI [0.005, 0.302], the indirect effect through the mediator of sociosexual orientation was also significant (*p* = 0.053), *b* = 0.051, SE = 0.027, 95% CI [0.003, 0.108], resulting in a significant total effect (*p* = 0.007), *b* = 0.196, SE = 0.073, 95% CI [0.062, 0.351]. These findings suggest that there is evidence for an association between childhood unpredictability and misperceptions of sexual interest. Here, we rely on confidence intervals rather than *p*-values to determine statistical significance, as bootstrapped confidence intervals provide a more robust test of mediation effects (Preacher and Hayes, 2008). Although the *p*-value is only marginally significant, sociosexual orientation likely plays a small but non-trivial mediating role.

For the pathway from childhood SES to misperceptions of sexual interest, mediated by sociosexual orientation, SES was not related to sociosexual orientation (*p* = 0.906), *b* = −0.005, SE = 0.045, 95% CI [−0.092, 0.084], but it had a significant direct effect on misperceptions of sexual interest (*p* = 0.022), *b* = 0.081, SE = 0.035, 95% CI [0.009, 0.149]. The indirect effect via sociosexual orientation was non-significant (*p* = 0.913), *b* = −0.001, SE = 0.006, 95% CI [−0.012, 0.013]. The total effect was significant (*p* = 0.024), *b* = 0.080, SE = 0.035, 95% CI [0.009, 0.149]. These findings are consistent with the results from the regression models, where the inclusion of sociosexual orientation did not reduce the effect of SES on misperceptions of sexual interest. Thus, childhood SES is directly and positively associated with misperceptions of sexual interest, independent of sociosexual orientation.

### 3.3 Discussion

Study 2 extends previous research by simultaneously examining the effects of childhood SES and environmental unpredictability. Regression analysis showed that childhood unpredictability was significantly associated with misperceptions of sexual interest in a workplace scenario involving unwanted sexual attention. Path analysis further suggested that unpredictability may be associated with misperceptions of sexual interest indirectly through unrestricted sociosexual orientation.

Interestingly, while SES was only marginally significant in the regression models regardless of whether sociosexual orientation was included, the direct effect of SES on misperceptions of sexual interest was stronger and statistically significant when sociosexual orientation was explicitly modelled as a mediator in the path model. This pattern may reflect a suppressor effect, where sociosexual orientation accounts for residual variance in misperceptions of sexual interest that is unrelated to SES. Therefore, when sociosexual orientation is included as a mediator, the “true” effect of SES on misperceptions of sexual interest is more accurately estimated, leading to a larger and statistically significant effect.

Contrary to our predictions, childhood SES was positively associated with misperceptions of sexual interest and perceived harassment. However, SES was not linked to the overall measure of sexual harassment perception because the reverse-coding of misperceptions of sexual interest (where lower mutual interest scores reflect greater recognition of sexual harassment) resulted in the associations offsetting one another. This led to a null effect for the composite measure of sexual harassment perception. These findings may be explained by the distinct socialisation processes associated with SES. Individuals from higher SES backgrounds may develop heightened social confidence, leading them to expect positive reciprocation in interactions and interpret scenarios more optimistically. Concurrently, their upbringing in relatively affluent families likely includes greater exposure to discussions about consent and personal boundaries, making them more vigilant in recognising behaviours that could constitute sexual harassment. This dual influence—optimism in interpreting social cues combined with the awareness of consent issues—may explain the seemingly contradictory associations between SES, misperceptions of sexual interest, and perceived harassment.

## 4 General discussion

This research provides novel cross-cultural evidence linking specific dimensions of ELA to mating-related behaviours, attitudes, and perceptions in adult men. While partial support for the life history framework was observed, the findings were mixed, with some effects diverging from its predictions. These results suggest that different dimensions of ELA may influence mating-related patterns through distinct mechanisms.

We found that higher childhood financial deprivation, used as a proxy of deprivation-based ELA, was positively associated with reproductive outcomes, including partner pregnancies and live births. This finding is consistent with predictions from the life history framework, which posits that individuals from deprived environments may adopt faster reproductive strategies in response to cues of environmental mortality risk. However, childhood SES was not associated with the number of sexual partners. This finding appears to contradict predictions from the life history framework. However, it aligns with the results of a recent meta-analysis, which illustrated that SES was not associated with the number of sexual partners in men (Xu et al., 2018), and was not linked to having more sexual partners at age 23 (Simpson et al., 2012). Previous work with women has also shown that higher childhood SES is associated with a greater number of sexual partners (Dinh et al., 2022). These findings suggest that SES encapsulates multiple constructs that might influence mating strategies in opposing ways. While low SES often reflects resource scarcity, which could promote an unrestricted mating strategy, resource abundance may facilitate unrestricted mating by enabling economic independence from stable partnerships. Furthermore, partner count may reflect broader social opportunities or relationship stability rather than reproductive strategy *per se*.

Interestingly, higher childhood SES was not related to unrestricted sociosexual orientation but was associated with stronger perceptions of both mutual romantic interest and sexual harassment in workplace contexts. While these findings may appear contradictory, they can be understood through the lens of cognitive socialisation, where cognitive functions develop through observational learning, imitation, and modelling, posited by social learning theory. Individuals from higher SES backgrounds may have greater access to education and more exposure to discussions about consent, increasing their sensitivity to violations of interpersonal boundaries (Tharumiya and Manicka, 2022). At the same time, they may be more likely to interpret ambiguous social cues as indicative of mutual interest due to heightened social confidence fostered by a higher SES environment. This dual socialisation process—optimism in social interactions combined with vigilance regarding social boundaries—could explain the simultaneous increase in misperceptions of sexual interest and perceived harassment. Alternatively, our findings seem to challenge the simplistic assumption of life history theory that lower childhood SES consistently promotes a quantity-over-quality reproductive strategy. Instead, when the environmental harshness is low, and resources are abundant relative to population size, selection may favour greater reproductive effort (Ellis et al., 2009). For example, In environments where resources like food and shelter are plentiful and competition is low, species may invest more in reproduction. From this perspective, by removing resource constraints, high SES may facilitate an adaptive shift towards increased reproductive effort. This underscores the need for more nuanced predictions within life history theory and highlights the importance of integrating frameworks that consider the effects of deprivation on cognitive development and moral reasoning.

Environmental unpredictability had a positive overall effect on perceptions of mutual romantic interest in a workplace scenario. This is consistent with life history theory, which suggests that growing up in unpredictable environments fosters mating strategies characterised by opportunistic mating and heightened sensitivity to social cues related to reproductive opportunities. Path analysis further revealed that unrestricted sociosexual orientation may mediate the association between unpredictability and misperceptions of sexual interest, although this effect was small. This suggests that individuals who grow up in unpredictable environments might be more likely to adopt unrestricted mating strategies and be more inclined to interpret social interactions as mutually romantic or positive. This pattern of results mirrors findings in the life history literature (e.g., Simpson et al., 2012; Szepsenwol et al., 2017; Xu et al., 2018).

However, unpredictability was not associated with the likelihood of interpreting unwanted sexual attention as harassment. This finding suggests that unpredictability affects mating-related perceptions, such as romantic interest, but not the cognitive recognition of harassment as a boundary violation. These judgements are likely shaped and better explained by factors beyond early environmental adversity, such as sociocultural influences. Specifically, harassment perception requires individuals to identify violations of interpersonal and legal norms, which are typically learned in formal, structured environments such as schools and workplaces. By contrast, unpredictability is associated with chaotic and inconsistent experiences. As such, unpredictability may have little impact on the cognitive processes required for recognising harassment.

The current studies provided mixed evidence for life history models, reflecting broader debates on their theoretical underpinnings. Critics argue that life history theory, originally developed to explain cross-species trait covariation, lacks a robust foundation for application to within-species differences (Stearns and Rodrigues, 2020; Zietsch and Sidari, 2020). While it remains a useful heuristic for understanding developmental influences on human variation, its theoretical assumptions require refinement, and its application would benefit from more rigorous methodologies. To enrich its explanatory power, it may be beneficial to incorporate complementary frameworks that account for the dimension-specific effects of ELA on mating strategies. One such framework is attachment theory, which posits that unstable parent-child bonding, often reflecting environmental unpredictability, fosters insecure attachment styles. These attachment patterns may manifest as unrestricted mating strategies, such as avoiding intimacy and adopting short-term mating (Fearon and Roisman, 2017). Supporting this perspective, research has shown that secure attachment was negatively associated with ELA (Lu et al., 2024), and partially mediates the relationship between early life predictability and restricted sociosexual orientation in adulthood (Szepsenwol et al., 2017).

To our knowledge, this research is among the first to examine how specific dimensions of ELA relate to sexual harassment perception. Our findings highlight distinct pathways: unpredictability appears to shape unrestricted sociosexual attitudes, increasing the likelihood of misinterpreting mutual interest, while lower SES might influence cognitive socialisation, leading to greater misidentification of boundary violations and coercion. Sexual harassment is a significant public health concern, that disproportionally affects women's wellbeing (Fitzgerald and Cortina, 2018; Wood et al., 2021). It has been linked to negative mental health outcomes (Ho et al., 2012; Sojo et al., 2016), substance abuse (Rospenda et al., 2008), and workplace consequences (O'Connell and Korabik, 2000). By exploring how ELA dimensions are associated with men's perceptions, this study contributes to a deeper understanding of the cognitive and social pathways underlying these perceptions.

## 5 Limitations and future directions

While our study provides key insights into how dimensions of ELA shape mating-related outcomes, several limitations should be noted. First, childhood unpredictability and SES were assessed via retrospective self-reports, which may introduce recall bias. Although subjective and objective SES are often correlated (Duncan et al., 2010), self-reports are susceptible to memory distortions. However, as the dimensional model of adversity suggests, subjective experience, rather than mere exposure, is what shapes psychological and behavioural adaptation (McLaughlin et al., 2021). To enhance measurement validity, future research should integrate both retrospective and objective indicators, such as parental income, parental occupation, or third-party reports (e.g., from parents or close relatives), to allow for direct comparisons. Second, genetic influences may confound the observed associations, as both shared genetic heritage and familial environments can shape sociosexual attitudes and social perceptions. Prior studies have found that the effects of early-life environments on adult traits are reduced or eliminated after controlling for genetic factors (Barbaro et al., 2017; Zietsch and Sidari, 2020). Future research should employ sibling or twin study designs to better isolate environmental effects and clarify the role of genetic influences. Third, while our findings indicate that childhood financial deprivation predicts higher reproductive outcomes in men, past research with women has reported null associations between SES and offspring count (Kometani and Ohtsubo, 2024; Richardson et al., 2024). This discrepancy highlights the need to explore potential sex differences in the effects of ELA on reproductive behaviour. In addition, future work should explore alternative explanatory mechanisms for the association between ELA and sexual harassment perceptions, such as sexism beliefs. For example, research suggests that harsher early environments may foster more traditional gender roles and sexist ideologies, which could, in turn, shape perceptions of sexual harassment (Zhu and Chang, 2019, 2020).

## 6 Conclusion

This research provides novel insights into how dimensions of ELA shape adult mating-related behaviours, attitudes, and perceptions. Deprivation-based adversity was associated with increased reproductive outcomes but not the number of sexual partners. Childhood SES was positively associated with perceptions of mutual interest and heightened recognition of boundary violations, potentially reflecting cognitive socialisation processes rather than evolutionary mechanisms. Unpredictability-based ELA was associated with misperceptions of sexual interest, likely through unrestricted sociosexual orientation, but was not associated with the recognition of boundary violations or harassment. These findings underscore the complexity of ELA effects, illustrating how deprivation and unpredictability shape adult outcomes through distinct psychological pathways. This research highlights the need to integrate and refine existing theories to better understand ELA's impact on human mating strategies.

## Data Availability

The Add Health data used in Study 1 is already published online and can be retrieved following the instructions provided by the Add Health Investigators (https://addhealth.cpc.unc.edu/data/). The dataset for study 2 can be found in the online repository linked here: https://osf.io/rfjt9/.

## Footnotes

^1^Although our study was initially pre-registered to include analyses involving life history strategy and sexual harassment proclivity measures, we decided to exclude these measures from the formal analyses. This decision was based on a careful re-evaluation of existing critiques in the literature, which question the validity and reliability of these scales in assessing the intended constructs. Notably, the results from these measures were largely consistent with our initial predictions; however, we opted to exclude them due to these methodological concerns. For transparency, these analyses are available upon request (the original pre-registration is available in OSF: https://osf.io/t672r).

## References

[B1] Barbaro N. Boutwell B. B. Barnes J. C. Shackelford T. K. (2017). Genetic confounding of the relationship between father absence and age at menarche. Evol. Hum. Behav. 38, 357–365. 10.1016/j.evolhumbehav.2016.11.00729978338

[B2] Belsky J. (2012). The development of human reproductive strategies: progress and prospects. Curr. Dir. Psychol. Sci. 21, 310–316. 10.1177/0963721412453588

[B3] Belsky J. Steinberg L. Draper P. (1991). Childhood experience, interpersonal development, and reproductive strategy: an evolutionary theory of socialization. Child Dev. 62:647. 10.2307/1131166 1935336

[B4] Dinh T. Haselton M. G. Gangestad S. W. (2022). “Fast” women? The effects of childhood environments on women's developmental timing, mating strategies, and reproductive outcomes. Evol. Hum. Behav. 43, 133–146. 10.1016/j.evolhumbehav.2021.12.001

[B5] Duncan G. J. Ziol-Guest K. M. Kalil A. (2010). Early-childhood poverty and adult attainment, behavior, and health. Child Dev. 81, 306–325. 10.1111/j.1467-8624.2009.01396.x 20331669

[B6] Ellis B. J. Figueredo A. J. Brumbach B. H. Schlomer G. L. (2009). Fundamental dimensions of environmental risk: the impact of harsh versus unpredictable environments on the evolution and development of life history strategies. Hum. Nat. 20, 204–268. 10.1007/s12110-009-9063-7 25526958

[B7] Ellis B. J. Sheridan M. A. Belsky J. McLaughlin K. A. (2022). Why and how does early adversity influence development? Toward an integrated model of dimensions of environmental experience. Dev. Psychopathol. 34, 447–471. 10.1017/S0954579421001838 35285791

[B8] Faul F. Erdfelder E. Lang A.-G. Buchner A. (2007). G^*^Power 3: a flexible statistical power analysis program for the social, behavioral, and biomedical sciences. *Behav. Res. Meth*. 39, 175–191. 10.3758/BF03193146 17695343

[B9] Fearon R. M. P. Roisman G. I. (2017). Attachment theory: progress and future directions. Curr. Opin. Psychol. 15, 131–136. 10.1016/j.copsyc.2017.03.002 28813253

[B10] Figueredo A. J. Vásquez G. Brumbach B. H. Schneider S. M. R. (2007). The K-factor, covitality, and personality: a psychometric test of life history theory. Hum. Nat. 18, 47–73. 10.1007/BF02820846 26181744

[B11] Fitzgerald L. F. Cortina L. M. (2018). “Sexual harassment in work organizations: a view from the 21st century,” in APA Handbook of the Psychology of Women: Perspectives on Women's Private and Public Lives, Vol. 2, eds. C. B. Travis, J. W. White, A. Rutherford, W. S. Williams, S. L. Cook, and K. F. Wyche (Washington, DC: American Psychological Association), 215–234. 10.1037/0000060-012

[B12] Fletcher G. J. O. Kerr P. S. G. Li N. P. Valentine K. A. (2014). Predicting romantic interest and decisions in the very early stages of mate selection: standards, accuracy, and sex differences. Pers. Soc. Psychol. Bull. 40, 540–550. 10.1177/0146167213519481 24501043

[B13] Griskevicius V. Tybur J. M. Delton A. W. Robertson T. E. (2011). The influence of mortality and socioeconomic status on risk and delayed rewards: A life history theory approach. *J. Pers. Soc. Psychol*. 100, 1015–1026. 10.1037/a0022403 21299312 PMC3298774

[B14] Gruijters S. L. K. Fleuren B. P. I. (2018). Measuring the unmeasurable: the psychometrics of life history strategy. Hum. Nat. 29, 33–44. 10.1007/s12110-017-9307-x 29143184 PMC5846862

[B15] Harris K. M. Halpern C. T. Whitsel E. A. Hussey J. M. Killeya-Jones L. A. Tabor J.. (2019). Cohort profile: the national longitudinal study of adolescent to adult health (add health). Int. J. Epidemiol. 48, 1415–1415k. 10.1093/ije/dyz115 31257425 PMC6857761

[B16] Haselton M. G. Buss D. M. (2000). Error management theory: a new perspective on biases in cross-sex mind reading. J. Pers. Soc. Psychol. 78, 81–91. 10.1037/0022-3514.78.1.81 10653507

[B17] Hilbe J. M. (2011). Negative Binomial Regression, 2nd Edn. Cambridge: Cambridge University Press. 10.1017/CBO9780511973420

[B18] Ho I. K. Dinh K. T. Bellefontaine S. A. Irving A. L. (2012). Sexual harassment and posttraumatic stress symptoms among Asian and White women. J. Aggress. Maltreat. Trauma 21, 95–113. 10.1080/10926771.2012.63323835960850

[B19] Howell E. C. Etchells P. J. Penton-Voak I. S. (2012). The sexual overperception bias is associated with sociosexuality. Pers. Individ. Dif. 53, 1012–1016. 10.1016/j.paid.2012.07.024

[B20] James J. Ellis B. J. Schlomer G. L. Garber J. (2012). Sex-specific pathways to early puberty, sexual debut, and sexual risk taking: tests of an integrated evolutionary–developmental model. Dev. Psychol. 48, 687–702. 10.1037/a0026427 22268605

[B21] Janicke T. Häderer I. K. Lajeunesse M. J. Anthes N. (2016). Darwinian sex roles confirmed across the animal kingdom. Sci. Adv. 2:e1500983. 10.1126/sciadv.1500983 26933680 PMC4758741

[B22] Klümper L. Schwarz S. (2020). Oppression or opportunity? Sexual strategies and the perception of sexual advances. *Evol. Psychol. Sci*. 6, 142–153. 10.1007/s40806-019-00215-y

[B23] Kohl C. Robertson J. (2014). The sexual overperception bias: an exploration of the relationship between mate value and perception of sexual interest. Evol. Behav. Sci. 8, 31–43. 10.1037/h0097247

[B24] Kometani A. Ohtsubo Y. (2024). Effects of accelerated reproductive timing in response to childhood adversity on lifetime reproductive success in modern environments. Evol. Psychol. Sci. 10, 240–249. 10.1007/s40806-024-00403-5

[B25] Långström N. Hanson R. K. (2006). High rates of sexual behavior in the general population: correlates and predictors. Arch. Sex. Behav. 35, 37–52. 10.1007/s10508-006-8993-y 16502152

[B26] Lacelle C. Hébert M. Lavoie F. Vitaro F. Tremblay R. E. (2012). Child sexual abuse and women's sexual health: the contribution of CSA severity and exposure to multiple forms of childhood victimization. J. Child Sex. Abus. 21, 571–592. 10.1080/10538712.2012.688932 22994694

[B27] Lee A. J. Sidari M. J. Murphy S. C. Sherlock J. M. Zietsch B. P. (2020). Sex differences in misperceptions of sexual interest can be explained by sociosexual orientation and men projecting their own interest onto women. Psychol. Sci. 31, 184–192. 10.1177/0956797619900315 31971873

[B28] Lu H. J. Lansford J. E. Liu Y. Y. Chen B. B. Bornstein M. H. Skinner A. T.. (2024). Attachment security, environmental adversity, and fast life history behavioral profiles in human adolescents. Dev. Psychopathol. 10.1017/S0954579424001500. [Epub ahead of print]. 39310941 PMC11929612

[B29] McGinnis E. W. Sheridan M. Copeland W. E. (2022). Impact of dimensions of early adversity on adult health and functioning: a 2-decade, longitudinal study. Dev. Psychopathol. 34, 527–538. 10.1017/S095457942100167X 35074038 PMC9309184

[B30] McLaughlin K. A. Sheridan M. A. Humphreys K. L. Belsky J. Ellis B. J. (2021). The value of dimensional models of early experience: thinking clearly about concepts and categories. Perspect. Psychol. Sci. 16, 1463–1472. 10.1177/1745691621992346 34491864 PMC8563369

[B31] McLaughlin K. A. Sheridan M. A. Lambert H. K. (2014). Childhood adversity and neural development: deprivation and threat as distinct dimensions of early experience. Neurosci. Biobehav. Rev. 47, 578–591. 10.1016/j.neubiorev.2014.10.012 25454359 PMC4308474

[B32] Mededović J. (2020). On the incongruence between psychometric and psychosocial-biodemographic measures of life history. Hum. Nat. 31, 341–360. 10.1007/s12110-020-09377-2 32918708

[B33] Mittal C. Griskevicius V. Simpson J. A. Sung S. Young E. S. (2015). Cognitive adaptations to stressful environments: When childhood adversity enhances adult executive function. *J. Pers. Soc. Psychol*. 109, 604–621. 10.1037/pspi0000028 26414842

[B34] Negriff S. Schneiderman J. U. Trickett P. K. (2015). Child maltreatment and sexual risk behavior: maltreatment types and gender differences. J. Dev. Behav. Pediatr. 36, 708–716. 10.1097/DBP.0000000000000204 26349071 PMC4635067

[B35] Norman R. E. Byambaa M. De R. Butchart A. Scott J. Vos T. (2012). The long-term health consequences of child physical abuse, emotional abuse, and neglect: a systematic review and meta-analysis. PLoS Med. 9:e1001349. 10.1371/journal.pmed.1001349 23209385 PMC3507962

[B36] O'Connell C. E. Korabik K. (2000). Sexual harassment: the relationship of personal vulnerability, work context, perpetrator status, and type of harassment to outcomes. J. Vocat. Behav. 56, 299–329. 10.1006/jvbe.1999.1717

[B37] Pedneault C. I. Babchishin K. M. Lalumière M. L. Seto M. C. (2020). The association between childhood sexual abuse and sexual coercion in men: a test of possible mediators. J. Sex. Aggress. 26, 193–211. 10.1080/13552600.2019.1613575

[B38] Penke L. Asendorpf J. B. (2008). Beyond global sociosexual orientations: a more differentiated look at sociosexuality and its effects on courtship and romantic relationships. J. Pers. Soc. Psychol. 95, 1113–1135. 10.1037/0022-3514.95.5.1113 18954197

[B39] Perilloux C. Easton J. A. Buss D. M. (2012). The misperception of sexual interest. Psychol. Sci. 23, 146–151. 10.1177/0956797611424162 22261567

[B40] Preacher K. J. Hayes A. F. (2008). Asymptotic and resampling strategies for assessing and comparing indirect effects in multiple mediator models. Behav. Res. Methods 40, 879–891. 10.3758/BRM.40.3.879 18697684

[B41] Ramiro L. S. Madrid B. J. Brown D. W. (2010). Adverse childhood experiences (ACE) and health-risk behaviors among adults in a developing country setting. Child Abuse Negl. 34, 842–855. 10.1016/j.chiabu.2010.02.012 20888640

[B42] Richardson G. B. Bates D. Ross A. Liu H. Boutwell B. B. (2024). Is reproductive development adaptively calibrated to early experience? Evidence from a national sample of females. Dev. Psychol. 60, 306–321. 10.1037/dev0001681 38190216

[B43] Rospenda K. M. Fujishiro K. Shannon C. A. Richman J. A. (2008). Workplace harassment, stress, and drinking behavior over time: gender differences in a national sample. Addict. Behav. 33, 964–967. 10.1016/j.addbeh.2008.02.009 18384975 PMC2442899

[B44] Senn T. E. Carey M. P. Vanable P. A. Coury-Doniger P. Urban M. A. (2006). Childhood sexual abuse and sexual risk behavior among men and women attending a sexually transmitted disease clinic. J. Consult. Clin. Psychol. 74, 720–731. 10.1037/0022-006X.74.4.720 16881780 PMC1578497

[B45] Shi X. Zheng Y. (2021). Feminist active commitment and sexual harassment perception among Chinese women: the moderating roles of targets' gender stereotypicality and type of harassment. *Sex Roles* 84, 477–490. 10.1007/s11199-020-01180-8

[B46] Simpson J. A. Griskevicius V. Kuo S. I.-C. Sung S. Collins W. A. (2012). Evolution, stress, and sensitive periods: the influence of unpredictability in early versus late childhood on sex and risky behavior. Dev. Psychol. 48, 674–686. 10.1037/a0027293 22329381

[B47] Sojo V. E. Wood R. E. Genat A. E. (2016). Harmful workplace experiences and women's occupational well-being: a meta-analysis. Psychol. Women Q. 40, 10–40. 10.1177/0361684315599346

[B48] Starrfelt J. Kokko H. (2012). Bet-hedging—a triple trade-off between means, variances and correlations. Biol. Rev. 87, 742–755. 10.1111/j.1469-185X.2012.00225.x 22404978

[B49] Stearns S. C. (1992). The Evolution of Life Histories. Oxford: Oxford University Press.

[B50] Stearns S. C. Rodrigues A. M. M. (2020). On the use of “life history theory” in evolutionary psychology. Evol. Hum. Behav. 41, 474–485. 10.1016/j.evolhumbehav.2020.02.001

[B51] Strouts P. H. Brase G. L. Dillon H. M. (2017). Personality and evolutionary strategies: the relationships between HEXACO traits, mate value, life history strategy, and sociosexuality. Pers. Individ. Dif. 115, 128–132. 10.1016/j.paid.2016.03.047

[B52] Szepsenwol O. Griskevicius V. Simpson J. A. Young E. S. Fleck C. Jones R. E. (2017). The effect of predictable early childhood environments on sociosexuality in early adulthood. Evol. Behav. Sci. 11, 131–145. 10.1037/ebs0000082

[B53] Tharumiya A. K. Manicka M. M. (2022). Gender as a predictor in the perception of sexual harassment definition. ECS Transac. 107, 16391–16396. 10.1149/10701.16391ecst20199521

[B54] Trivers R. (1972). “Parental investment and sexual selection,” in Sexual Selection and the Descent of Man, 1871-1971, ed. B. Campbell (Chicago, IL: Aldine), 378.

[B55] Wang J. Chen B.-B. (2016). The influence of childhood stress and mortality threat on mating standards. Acta Psychol. Sini. 48:857. 10.3724/SP.J.1041.2016.0085737113526

[B56] Wood L. Hoefer S. Kammer-Kerwick M. Parra-Cardona J. R. Busch-Armendariz N. (2021). Sexual harassment at institutions of higher education: prevalence, risk, and extent. J. Interpers. Viol. 36, 4520–4544. 10.1177/0886260518791228 30071790 PMC10676016

[B57] Xu Y. Norton S. Rahman Q. (2018). Early life conditions, reproductive and sexuality-related life history outcomes among human males: a systematic review and meta-analysis. Evol. Hum. Behav. 39, 40–51. 10.1016/j.evolhumbehav.2017.08.005

[B58] Yang A. Zhu N. Lu H. J. Chang L. (2022). Environmental risks, life history strategy, and developmental psychology. PsyCh J. 11, 433–447. 10.1002/pchj.561 35599317

[B59] Young E. S. Griskevicius V. Simpson J. A. Waters T. E. A. Mittal C. (2018). Can an unpredictable childhood environment enhance working memory? Testing the sensitized-specialization hypothesis. *J. Pers. Soc. Psychol*. 114, 891–908. 10.1037/pspi0000124 29389153

[B60] Yuan J. Yu Y. Liu D. Sun Y. (2022). Associations between distinct dimensions of early life adversity and accelerated reproductive strategy among middle-aged women in China. Am. J. Obstetr. Gynecol. 226, 104.e1-104.e14. 10.1016/j.ajog.2021.07.033 34384774

[B61] Zhu N. Chang L. (2019). Evolved but not fixed: a life history account of gender roles and gender inequality. Front. Psychol. 10:1709. 10.3389/fpsyg.2019.01709 31396136 PMC6664064

[B62] Zhu N. Chang L. (2020). An evolutionary life history explanation of sexism and gender inequality. Pers. Individ. Dif. 157:109806. 10.1016/j.paid.2019.109806

[B63] Zietsch B. P. Sidari M. J. (2020). A critique of life history approaches to human trait covariation. Evol. Hum. Behav. 41, 527–535. 10.1016/j.evolhumbehav.2019.05.007

